# Modulation of Host Biology by *Pseudomonas aeruginosa* Quorum Sensing Signal Molecules: Messengers or Traitors

**DOI:** 10.3389/fmicb.2015.01226

**Published:** 2015-11-09

**Authors:** Yi-Chia Liu, Kok-Gan Chan, Chien-Yi Chang

**Affiliations:** ^1^Division of Molecular Microbiology, School of Life Sciences, University of DundeeDundee, UK; ^2^Division of Genetics and Molecular Biology, Institute of Biological Sciences, Faculty of Science, University of MalayaKuala Lumpur, Malaysia; ^3^Centre for Bacterial Cell Biology, Medical School, Newcastle UniversityNewcastle upon Tyne, UK; ^4^Interdisciplinary Computing and Complex BioSystems (ICOS) Research Group, School of Computing Science, Newcastle UniversityNewcastle upon Tyne, UK

**Keywords:** Quorum sensing, *N*-acyl homoserine lactones, *Pseudomonas* quinolone signal, *Pseudomonas aeruginosa*, immunomodulation

## Abstract

Bacterial cells sense their population density and respond accordingly by producing various signal molecules to the surrounding environments thereby trigger a plethora of gene expression. This regulatory pathway is termed quorum sensing (QS). Plenty of bacterial virulence factors are controlled by QS or QS-mediated regulatory systems and QS signal molecules (QSSMs) play crucial roles in bacterial signaling transduction. Moreover, bacterial QSSMs were shown to interfere with host cell signaling and modulate host immune responses. QSSMs not only regulate the expression of bacterial virulence factors but themselves act in the modulation of host biology that can be potential therapeutic targets.

## Introduction

Quorum sensing (QS) is coined to describe the phenomenon of an intercellular co-operative behavior of bacteria used to coordinate the activities of individual cells. Diffusible QS signal molecules (QSSMs) play crucial roles in signal transduction of which, when QSSMs reach a threshold concentration, can coordinate multiple gene expression and a change in the behavior of bacterial population through the activation of sensor regulatory proteins (Fuqua et al., 1994; Miller and Bassler, 2001; Williams and Cámara, 2009). Bacteria produce a broad-range of signal molecules. Different types of QSSMs have been identified and characterized (LaSarre and Federle, 2013). Besides prokaryote, bacterial QSSMs also affect the settlement and germination of eukaryotic seaweed zoospores (Joint et al., 2002; Twigg et al., 2013). In several pathogenic bacteria, QS control their virulence determinants and contribute to bacterial pathogenesis. Due to the fact that the population density-dependent regulatory systems used by many bacterial pathogens are not essential for survival under most conditions, the disruption/interference of QS is considered an alternative approach to attenuate bacterial virulence in infections (LaSarre and Federle, 2013). However, this point has recently been argued that the resistance mechanisms against QS inhibitors could be possible and have been identified (Defoirdt et al., 2010, 2013; García-Contreras et al., 2013, 2015a).

*Pseudomonas aeruginosa* is an ubiquitous Gram-negative bacterium with remarkably large and complex genome and is capable of adapting to versatile environments. In human cystic fibrosis (CF) lungs where *P. aeruginosa* has evolved the ability to form biofilms which are difficult to be eradicated by antibiotics (Heeb et al., 2011; Winsor et al., 2011). QS is responsible for the regulation of a large number of genes, for instance, around 10% of genes in the genome of *P. aeruginosa* are regulated by QS (Williams and Cámara, 2009). Here we review recent advances of *P. aeruginosa* QSSMs focusing on their roles in interference with host cells (**Table 1**) and the development of novel compounds that counteract the QSSMs activities.

**Table 1:** Bacterial quorum sensing molecules and their roles in pathogenesis/immunomodulation.

Quorum sensing (QS) signal molecules (QSSMs)	Mechanism of virulence	Effect concentrations	Reference
**3-oxo-C12-HSL**	**Apoptosis and cytotoxicity**		
	3-oxo-C12-HSL is cytotoxic to murine bone-marine derived macrophages, neutrophils and monocytic cell lines.	12–50 μM	Tateda et al., 2003
	3-oxo-C12-HSL induces apoptosis in murine fibroblasts and human vascular endothelial cells (HUVEC)	100 μM	Shiner et al., 2006
	3-oxo-C12-HSL triggers intrinsic apoptotic pathway in airway epithelial cells including depolarization of mitochondrial membrane potential, release of cytochrome C and activation of caspases 3/7, 8, and 9. 3-oxo-C12-HSL-mediated apoptosis is independent of the presence of CFTR in airway epithelial cells	>10 μM	Schwarzer et al., 2010
	3-oxo-C12-HSL promotes human mesenchymal stem cells (MSCs) apoptosis	50 μM	Holban et al., 2014
	**Disruption of barrier integrity**		
	*Pseudomonas aeruginosa* 3O-C(12)-HSL causes the loss of epithelial barrier function via calcium signaling and further alteration in the phosphorylation status of junction proteins	20–200 and 10 μM with slower response	Vikström et al., 2009, 2010
	**Immunomodulation and/or signaling**		
	3-oxo-C12-HSL promotes the expression and production of IL-8 in human epithelial and fibroblast cells through the induction of NF-kB via the phosphorylation of ERK/MARK	100 μM	Smith et al., 2001
	3-oxo-C12-HSL inhibits ConA-activated PBMCs proliferation and IL-2 secretion	^a^IC_50_: 18.24 μM	Hooi et al., 2004
	3-oxo-C12-HSL inhibits the proliferation of anti-CD3/anti-CD28 antibody activated T cells	IC_50_: 44.47 μM	Hooi et al., 2004
	3-oxo-C12-HSL inhibits the differentiation of Th1 and Th2 cells	5 μM	Ritchie et al., 2005
	3-oxo-C12-HSL increases the cytosolic calcium levels and calcium release through inositol triphosphate (IP3) receptors in the ER.	1 mM	Shiner et al., 2006
	3-oxo-C12-HSL promotes neutrophil chemotaxis, phagocytosis and up-regulates the expression of CD11b/CD18 and CD16/CD64 receptors	100 μM	Zimmermann et al., 2006; Wagner et al., 2007
	3-oxo-C12-HSL selectively disrupts NF-κB signaling but not TLR-dependent pathways in activated macrophages	50 μM	Kravchenko et al., 2006, 2008
	3-oxo-C12-HSL binds to PPARγ ligand binding domain	25–50 μM	Jahoor et al., 2008; Cooley et al., 2010
	3-oxo-C12-HSL increases the secretion of IL-1β in human MSCs	50 μM	Holban et al., 2014
	3-oxo-C12-HSL activates NF-κB p65 by preventing the re-synthesis of IκB, increases transcription of KC and IL-6 but inhibits secretion of KC and IL-6 by MEFs. 3-oxo-C12-HSL activates PERK and inhibits protein synthesis	50 or 100 μM	Grabiner et al., 2014
**Alkylquinolones**	**Change of bacterial behaviors**		
	Bacterial autolysis	Spent culture supernatant 15 μg on filter discs	Williams and Cámara, 2009
	Iron chelation	50 μM	Diggle et al., 2007
	exDNA release	Genetic and phenotype study	Allesen-Holm et al., 2006
	Oxidative functions	>100 μM *in vitro*	Häussler and Becker, 2008
	**Apoptosis**		
	HHQ promotes human MSCs apoptosis	50 μM	Holban et al., 2014
	**Immunomodulation and/or signaling**		
	*Pseudomonas* quinolone signal (PQS) inhibits the proliferation of ConA-activated PBMCs	IC_50_: 0.90 μM	Hooi et al., 2004
	PQS affects IL-2 secretion of ConA-stimulated PBMCs	IC_50_: 2.03 μM	Hooi et al., 2004
	PQS promotes TNF-α production in LPS-treated monocytes	>25 uM	Hooi et al., 2004
	Inhibition of IL-12 production in dendritic cells resulting in reduction of T-cell proliferation	IC_50_: 17.2 μM	Skindersoe et al., 2009
	Inhibition of NF-κB and HIP-1α pathways in murine epithelial cells and murine macrophages	Bacterial culture supernatants approx. PQS in PA14, 15 μM approx. PQS in *pqsL*, 45 μM	Kim et al., 2010; Legendre et al., 2012
	Stimulation of chemotaxis of neutrophils via the MAPK and p38 signaling pathways	10–100 μM	Hänsch et al., 2014
	PQS activates the secretion of IL-6 and HHQ induces IL-10 secretion by human MSCs	50 μM	Holban et al., 2014

*^a^50% inhibitory concentration (IC_50_).*

## *N*-Acyl Homoserine Lactones (AHLS) and their Modulations in Host Cells

Gram-negative bacteria, like *Aliivibrio fischeri* (previous *Vibrio fischeri*; Urbanczyk et al., 2007), have a conserved QS system with two central components, the LuxR-type and LuxI-type proteins, which serve as the signal receptor and signal synthase, respectively. LuxI catalyzes the synthesis of signaling molecules called *N*-acyl homoserine lactones (AHLs). When an AHLs concentration of 10 nM is reached, AHLs interact with LuxR and form a complex which promotes the expression of target genes, *luxICDABE* for bioluminescence production and also the LuxI production (Kaplan and Greenberg, 1985). This forms a positive loop to produce more signal molecules (Fuqua et al., 1994; Cámara et al., 2002; Fuqua and Greenberg, 2002). The *N*-acyl homoserine lactone consists of a homoserine lactone ring from *S*-adenosylmethionine (SAM) and acyl chain from acyl acyl-carrier-protein (acyl-ACP) linked by an amide bond (Parsek et al., 1999). Based on the acyl-ACP binding site, different LuxI homologs produce different AHLs with various acyl side chains (Watson et al., 2002; Gould et al., 2004). A broad range of AHLs is produced in Gram-negative bacteria and AHL-QS systems control various bacterial behaviors (LaSarre and Federle, 2013). In *A. fischeri N*-(3-oxohexanoyl) homoserine lactone (3-oxo-C6-HSL) is produced for controlling bioluminescence production. In *P. aeruginosa* two AHL synthases, RhlI and LasI, produce a wide spectrum of AHLs including *N*-butanoyl-homoserine lactone (C4-HSL), *N*-hexanoyl-homoserine lactone (C6-HSL) by RhlI and *N*-(3-oxooctanoyl)-homoserine lactone (3-oxo-C8-HSL), *N*-(3-oxodecanoyl)-homoserine lactone (3-oxo-C10-HSL), *N*-(3-oxododecanoyl)-homoserine lactone (3-oxo-C12-HSL) and *N*-(3-oxotetradecanoyl)-homoserine lactone (3-oxo-C14-HSL) by LasI (Ortori et al., 2011). An unusual *N*-(3-oxohexadecanoyl)-homoserine lactone (3-oxo-C16-HSL) secreted by an environmental *Pseudomonas* sp. from a diseased Tilapia fish suggests that 3-oxo-C16-HSL may contribute to the pathogenesis (Chang et al., 2012).

The abundant concentration of 3-oxo-C12-HSL in the culture of *P. aeruginosa* prompted investigations for its role in the pathogenesis with a mechanism potentially distinct from other pathogens. Indeed, 3-oxo-C12-HSL was found to activate mammalian cells through a mechanism independent of the toll-like receptor (TLR) pathways (Kravchenko et al., 2006). 3-oxo-C12-HSL was shown to activate pro-inflammatory responses in human epithelial and fibroblast cells through the induction the transcriptional factor, nuclear factor kappa-light-chain-enhancer of activated B cells (NF-κB) via the phosphorylation of ERK/MARK (Smith et al., 2001). However, this molecule selectively disrupts the NF-κB signaling pathway in activated macrophages (Kravchenko et al., 2008). Studies indicated that 3-oxo-C12-HSL not only induces apoptosis in haematopoietic cells but is cytototoxic to non-haematopoietic cells including airway epithelial cells, endothelial cells, fibroblasts, and mesenchymal stem cells (Tateda et al., 2003; Shiner et al., 2006; Schwarzer et al., 2010, 2012; Grabiner et al., 2014; Holban et al., 2014). *P. aeruginosa* 3-oxo-C12-HSL also impairs the epithelial barrier integrity through the alternations of calcium signaling and phosphorylation status of junctional proteins in the intestinal epithelial cells (Vikström et al., 2009, 2010).

In addition to its cytotoxicity, the role of 3-oxo-C12-HSL in immunomodulation has been intensively investigated (**Table 1**). Ritchie et al. (2005) reported that 3-oxo-C12-HSL inhibits the differentiation of Th1 and Th2 cells. Human polymorphonuclear neutrophils (PMNs) are attracted by 3-oxo-C12-HSL and increasingly express the adhesion proteins CD11b/CD18 and the immunoglobulin receptors CD16 and CD64 (Zimmermann et al., 2006; Wagner et al., 2007). The downregulation of the immune responses by 3-oxo-C12-HSL was demonstrated in human monocytes and murine macrophage-like cells in the presence of lipopolysaccharides (LPS) that 3-oxo-C12-HSL inhibits the production of pro-inflammatory cytokine tumor necrosis factor α (TNF-α) but promotes the production of anti-inflammatory cytokine interleukin-10 (IL-10; Hooi et al., 2004; Glucksam-Galnoy et al., 2013). Grabiner et al. (2014) noticed that despite the increasing transcriptional expression of the murine interleukin 8 (IL-8) homologs KC and interleukin 6 (IL-6) in murine embryonic fibroblasts (MEFs), KC and IL-6 protein secretion were inhibited by the treatment of 3-oxo-C12-HSL. It was shown that 3-oxo-C12-HSL acts upon the activation of endoplasmic reticulum (ER) stress transducer protein kinase RNA-like ER kinase (PERK) leading to the inhibition of protein synthesis. However, PERK is independent of 3-oxo-C12-HSL induced apoptosis indicating that 3-oxo-C12-HSL interferes with host cell biological activities through different mechanisms (Grabiner et al., 2014). Recent advances on the interactions between 3-oxo-C12-HSL and various types of host cells are highlighted in the review (Holm and Vikström, 2014).

**FIGURE 1:**
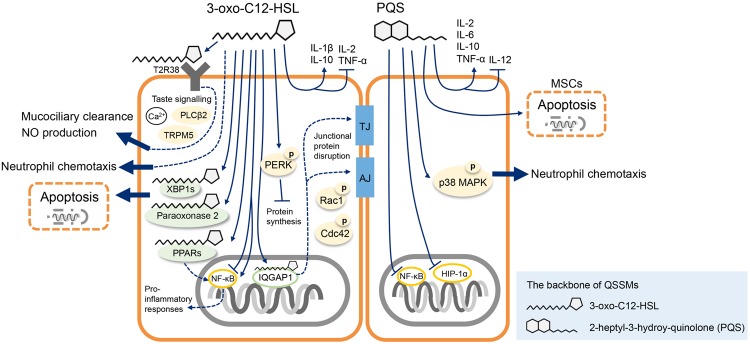
**Schematic illustration of the best characterized *Pseudomonas aeruginosa* quorum sensing signal molecules (QSSMs) that interfere with mammalian host biological functions.** *P. aeruginosa* 3-oxo-C12-HSL targets XBP1 and paraoxonase 2 leading to host cell apoptosis, and the binding of 3-oxo-C12-HSL with IQGAP1 impairs the host cell integrity. 3-oxo-C12-HSL modulates innate immune responses via the activation of T2R38 receptor and inhibition of NF-κB pathways. *P. aeruginosa Pseudomonas* quinolone signal (PQS) molecule induces MSCs apoptosis, induces the secretion of inflammatory cytokines via the inhibition NF-κB and HIP-1α pathways. PQS molecule interferes with neutrophils chemotaxis potentially through the activation of p38 MAPK pathways. See text for details. PLCβ2, phospholipase C β2; TRPM5, transient receptor potential cation channel subfamily M member 5; PPARs, peroxisome proliferator-activated receptors; TJ, tight junction; AJ, adhesional junction; MAPK, mitogen-activated protein kinase; MSCs, mesenchymal stem cells.

Several host targets of 3-oxo-C12-HSL have been identified (**Figure 1**). In murine fibroblasts and human lung epithelial cells peroxisome proliferator-activated receptor beta/delta (PPARβ/δ) and PPARγ may be the 3-oxo-C12-HSL receptors for pro-inflammatory responses (Jahoor et al., 2008; Cooley et al., 2010). 3-oxo-C12-HSL interacts and co-localizes with the IQ-motif-containing GTPase-activating protein IQGAP1 in human intestinal epithelial cells that causes the alternation of cell migration in a Rac1 and Cdc42- dependent manner (Karlsson et al., 2012). MEFs in lack of a transcriptional factor X-box binding protein 1 transcription factor (XBP1s) are protective from 3-oxo-C12-HSL and C14-HSL (*N*-tetradecanoyl-homoserine lactone) mediated apoptosis indicating that XBP1s is a critical host target in response of AHLs (Valentine et al., 2013). Paraoxonase 2, in response to 3-oxo-C12-HSL through its lactonase activity, leads to apoptosis in human and murine embryotic epithelial cells (Schwarzer et al., 2015). Interestingly, 3-oxo-C12-HSL activates the expression of a taste receptor T2R38 on the surface of primary human sinonasal cells (Lee et al., 2014) and neutrophils (Maurer et al., 2015). This recognition regulates calcium-dependent NO production thereby stimulates the mucociliary clearance and antibacterial effects suggesting an alternative innate immune defense mechanism distinct from the activation by canonical pattern recognition receptors (PRRs; Lee et al., 2012, 2014). Identification of the host compartments targeted by QSSMs could be the milestone for developing effective therapeutic methods against infections.

## Pathogenic Roles of Alkyl-Quinolone Signals

*Pseudomonas aeruginosa* also employs the alkyl-quinolone (AQ)-based QS system and the signal molecule was termed *Pseudomonas* quinolone signal (PQS; Pesci et al., 1999). The study of the AQs began from their intriguing structures similar to antimicrobial quinolones, although AQs were found no antimicrobial activities. Further studies unveiled that among more than 50 alkyl-quinolones found in *P. aeruginosa*, 2-heptyl-3-hydroxy-4-(1H)-quinolone (PQS) and its precursor molecular 2-heptyl-4(1*H*)-quinolone (HHQ) are major QSSMs that cooperates with the AHL-QS (Xiao et al., 2006; Heeb et al., 2011). Synthesis of PQS depends on the *pqsABCDE* operon. PqsA, the anthranilate co-enzyme A ligase, catalyzes anthranilate that is produced by PhnAB to anthraniloyl-coenzyme A. PqsD mediates the synthesis of 2-aminobenzoylacetate (2-ABA) from anthraniloyl-coenzyme A and malonyl-CoA, decarboxylating coupling of 2-ABA to an octanoate group of octanoic acid that linked to PqsBC to produce HHQ (Dulcey et al., 2013). A recent study suggested PqsE is involved in the HHQ synthesis through hydrolysing the 2-ABA-CoA to form 2-ABA (Drees and Fetzner, 2015). HHQ can be transformed to PQS by the mono-oxygenase PqsH encoded by *pqsH* located elsewhere on the chromosome (Pesci et al., 1999; Diggle et al., 2006). PqsR, also known as MvfR, is a LysR-type transcriptional regulator, with a conserved N-terminal DNA-binding helix-turn-helix and a C-terminal co-inducer-binding domain. PqsR activates the transcription of *pqsABCDE* and possibly the *phnAB* operon when binding to PQS or HHQ and triggers the typical QS autoinducing response enhancing AQ biosynthesis (Maddocks and Oyston, 2008; Heeb et al., 2011). PQS has been shown to reach the maximal production at late logarithmic phase (Diggle et al., 2003) and its production is promoted by the availability of the substrate anthranilate and the presence of aromatic amino acids (Palmer et al., 2005). AQ- and AHL-QS in *P. aeruginosa* are hierarchical and involved in the regulation of multiple virulence factors including rhamnolipids, pyocyanin, elastases, exotoxin A, and alkaline protease (Xiao et al., 2006; Dubern and Diggle, 2008; Nadal Jimenez et al., 2012).

*Pseudomonas* quinolone signal is considered a multifunctional molecule. PQS is involved in bacterial cell autolysis at high population densities in nutrient deprived conditions (Williams and Cámara, 2009). PQS also has iron-chelating properties that contribute to iron transport and facilitates siderophore-mediated iron delivery (Diggle et al., 2007). It has been demonstrated that there is far less extracellular DNA (exDNA) released by a *pqsA* mutant than its wild-type counterpart either in planktonic or biofilm cultures (Allesen-Holm et al., 2006). PQS has dual pro- and anti-oxidative functions for developing different levels of tolerance in *P. aeruginosa* cells to environmental stress (Häussler and Becker, 2008). This may shape the whole population structure, increase the fitness in hostile environments and lead to the development of resistance to host immune systems (García-Contreras et al., 2015a,b).

A new QS molecule, 2-(2-hydroxyphenyl)-thiazole-4-carbaldehyde (IQS) encoded by the *ambBCDE* operon was discovered recently (Lee et al., 2013). IQS is induced when *P. aeruginosa* is exposed to a phosphate-deprived environment. Under this unfavorable environment, expression of IQS overcomes the *las*-led QS circuit and promotes the expression of virulence factors. This finding may partially explain how *P. aeruginosa* clinical isolates persist in CF respiratory infections in the absence of a functional *las* system. Despite the fact that the AHL-QS of *P. aeruginosa* have been shown to play central roles in the regulation of virulence and immune modulation *in vitro*, this situation could differ *in vivo*. Around 50% of strains isolated from lungs of late stage CF patients are deficient in *lasR* function (Winstanley and Fothergill, 2009). Moreover, abolishing the whole AHL-QS by generating a quadruple mutation of *rhlIR* and *lasIR* exerted comparable infectivity to the wild-type strain in a mouse lung infection model (Lazenby et al., 2013), suggesting that the AHL-QS may not be required for full pathogenesis *in vivo* and other regulatory mechanism could be involved.

The contribution of the AQ-QS system to *P. aeruginosa* virulence was firstly described by Cao et al. (2001). PqsR positively regulates the expression of *phnAB* operon and the production of elastase, 3-oxo-C12-HSL and PQS that promotes the production of numerous virulence determinants. The *pqsR* mutant was attenuated up to 320-fold in the *Arabidopsis* plant infection model and caused a 65% reduction of mortality in a murine burn wound model (Cao et al., 2001). The interaction of PqsR with the AHL-QS was investigated by other groups showed that the effect of *pqsR* deficiency on pathogenesis is independent from LasRI/RhlRI (Déziel et al., 2004; Dubern and Diggle, 2008). Mutations in the multidrug eﬄux pump, such as *mexI* and *opmD* led to the inhibition of PQS production and the attenuation of *P. aeruginosa* in rat and plant infection models. Provision of exogenous AQs to these mutants restored the virulence on plants (Aendekerk, 2005). Rampioni et al. (2010) found that both *pqsA* and *pqsE* mutants in PAO1 were attenuated in plant, nematode and mouse burn wound infection models. In an acute urinary tract infection model, PQS molecules were present in the renal and bladder tissue of mice infected with wild-type *P. aeruginosa* but absent in the mice with PQS mutants infections (Bala et al., 2014). Wild-type *P. aeruginosa* caused more severe inflammation and tissue destruction and greater levels of inflammatory cytokines TNF-α, IL-6, and IL-10 at the site of infection in mice infected with wild-type strain than with PQS mutants. The virulence of PQS mutants can be restored by the addition of exogenous PQS molecules (Bala et al., 2014). These findings indicate that PQS participates in the pathogenesis of *P. aeruginosa*.

*Pseudomonas* quinolone signal has been identified in sputum, bronchoalveolar lavage fluid (BAL) and mucopurulent fluid from distal airways of end-stage CF lungs removed for transplant and at different stages from asymptotic early stage to late progression, suggesting a potential role of PQS in coordinating virulence factors during the course of infections (Collier et al., 2002; Guina et al., 2003). A study involving 60 CF patients with chronic *P. aeruginosa* infection indicated that the AQs were detectable in the sputum, plasma and urine and the concentrations of molecules are positively correlated to the *P. aeruginosa* bacterial cell density. 2-nonyl-4-hydroxy-quinoline (NHQ) in plasma was suggested to be the biomarker for *P. aeruginosa* infection in CF lungs (Barr et al., 2015). An *in vitro* transcriptomic study investigating the physiology of *P. aeruginosa* grown in CF sputum revealed that the genes associated with PQS metabolism, such as those coding for the aromatic amino acid aminotransferase, 4-hydroxyphenylpyruvate dioxygenase (*hpd*) and *pqsABCDE*, were expressed 10-fold greater than the expression when *P. aeruginosa* was cultured in media containing glucose alone as the carbon source (Palmer et al., 2005).

*Pseudomonas aeruginosa* AQ molecules have been implicated in the immuno-modulation on host cells. PQS was shown to modulate cell proliferation, the production of interleukin-2 (IL-2) and TNF-α in mitogen-stimulated human peripheral blood mononuclear cells (PBMCs; Hooi et al., 2004). PQS inhibited the production of IL-12 by LPS-stimulated bone marrow-derived dendritic cells which led to reduced T-cell proliferation (Skindersoe et al., 2009). Additionally AQ extracts derived from *P. aeruginosa* PA14 supernatants down-regulated host innate immune responses via inhibition of the NF-κB and hypoxia-inducible factor 1 alpha (HIF-1α) pathways in murine macrophages and cells obtained from BAL (Kim et al., 2010; Legendre et al., 2012). A recent study addressed the importance of timing in neutrophil infiltration in relation to the role of PQS in interference with neutrophil chemotaxis. Low levels of PQS stimulated the chemotaxis of neutrophils via the MAPK and p38 signaling pathways, whereas high levels of PQS, most likely produced by biofilm-like *P. aeruginosa*, did not interfere with neutrophils phagocytic capability and viability (Hänsch et al., 2014). Massive neutrophil accumulation is commonly seen in CF airways and high levels of neutrophil elastase correlate with poor pulmonary functions (Downey et al., 2008; Gifford and Chalmers, 2014). PQS may thus provide *P. aeruginosa* with another strategy for bacterial survival via the interference in multiple aspects of host biological activities.

## The Development of Inhibitors and Vaccines Against QSSMS

Due to the fact that QSSMs have been implicated in the involvement of pathogenesis, the search for inhibitors and the development of vaccines that antagonize QSSMs are currently intensively investigated. Chang et al. (2014) suggested a strategy to screen novel anti-QS compounds from plant extraction that potentially could tackle the QS-mediated infections. The antibody 3-oxo-C12-HSL-BSA conjugate was also shown to alleviate the inflammatory responses by *P. aeruginosa* infections in an acute murine lung infection model (Miyairi et al., 2006). In a burn wound infection model, mice immunized with the vaccine 3-oxo-C12-HSL-r-PcrV conjugate before *P. aeruginosa* infection had higher survival than those without immunization (Golpasha et al., 2015). A high-throughput screening approach based on the inhibition of C12-mediated host responses identified triazolo[4,3-a]quinolines as 3-oxo-C12-HSL inhibitors with nanomolar potency that restore NF-κB activity in 3-oxo-C12-HSL treated cell lines and shown protective using an *in vivo* dermal infection model (Valentine et al., 2014). Since anthranilate (AA) being the precursor of AQs, halogenated AA analogs were found to inhibit the AQ biosynthesis and down-regulate the expression of PqsR controlled genes. Treatment with AA analogs prior to *P. aeruginosa* infection increased mice survival and lowered the bacterial dissemination to the organs (Lesic et al., 2007). MvfR-regulon inhibitors that bind QS transcriptional regulator MvfR (PqsR) were not only protective in murine acute and persistent infections against *P. aeruginosa* but also effectively reduced the formation of antibiotic-tolerant persisters (Starkey et al., 2014). These studies suggest the therapeutic potential of inhibitors and vaccines against QSSMs in both acute and chronic infections.

## Conclusion

Quorum sensing-based bacterial communication links the individual bacterial cells to behave as multicellular organisms by employing signal molecules and to promote its population survival in the environment or hosts. QSSMs also interact with host cell signal pathways and the modulation of immune cell biology. For more than a decade strategies have been proposed from the use of inhibitors of QS for containing chronic infections (Hentzer et al., 2003) to the application of QSSMs for modulating immune responses to bacterial infections (Hancock et al., 2012). Understanding how QSSMs interact with host cells seems the promising land to tackle bacterial infections. Here we discussed recent advances on the interference of QSSMs with mammalian cells, the recently identified receptors on mammalian cells that target QSSMs and the QSSM inhibitors and their mechanisms. However, contradictory results suggested that many unknown mechanisms in complex bacteria-host interactions are remained. Also concern about bacterial resistance to QS inhibitors (García-Contreras et al., 2015a) and unexpected impact of QS inhibitors to environment (Decho et al., 2010) is increasing. Extending our understanding of the multiple roles of QSSMs would be valuable in the development of new therapeutic strategies against bacterial infections.

## Author Contributions

Y-CL, K-GC, C-YC wrote the paper. Y-CL made the figure. Y-CL and C-YC made the table.

## Conflict of Interest Statement

The authors declare that the research was conducted in the absence of any commercial or financial relationships that could be construed as a potential conflict of interest.
